# Prevalence and patterns of e-cigarette use among school-aged adolescents in Peru, 2024

**DOI:** 10.18332/tid/218168

**Published:** 2026-06-05

**Authors:** Rodrigo Vargas-Fernández, Akram Hernández-Vásquez

**Affiliations:** 1 Epidemiology and Health Economics Research (EHER), Universidad Científica del Sur, Lima, Peru; 2 Centro de Excelencia en Investigaciones Económicas y Sociales en Salud, Vicerrectorado de Investigación, Universidad San Ignacio de Loyola, Lima, Peru

**Keywords:** electronic nicotine delivery systems, cigarette smoking, e-cigarette vapor, prevalence, Peru

## Abstract

**INTRODUCTION:**

The rapid rise in e-cigarette use among adolescents has become a global public health concern. Although regional data from Latin America exist, Peru lacks national evidence. We aimed to estimate the prevalence, describe patterns and explore motivations for e-cigarette use among secondary school students.

**METHODS:**

We conducted a secondary data analysis of the 2024 National survey on drug use in secondary school students of Peru. The survey was administered through questionnaires to 45345 students from grades 1–5 of secondary education in urban areas. Weighted prevalences of ever, past 12-month, current and dual use (concurrent use of e-cigarettes and conventional cigarettes) were estimated. Analyses were conducted by sex and sources of access and motivations for use were also assessed.

**RESULTS:**

We included 45089 adolescents. Girls initiated e-cigarette use earlier than boys [age 14.7 (SD=1.3) vs 15.0 (SD=1.4) years, p<0.001]. The prevalence of ever use was 16.0% (95% CI: 15.5–16.6), higher in girls than boys (17.1% vs 15.0%, p<0.001). Past 12-month use was 10.9% (95% CI: 10.5–11.3), mostly 1–5 days, while current use was 6.9% (95% CI: 6.6–7.3) with no sex differences. Dual use was reported by 5.1% in the past 12 months and 2.7% in the past 30 days, the latter more frequent in boys (p=0.005). Moreover, the proportion of adolescents reporting each outcome increased progressively from first to the fifth grade. The main sources of access were convenience stores (29.7%) and neighborhood shops (18.1%). The leading motivations for use were flavor (30.8%), curiosity (20.4%) and stress management (12.9%). Differences in motivations were evident by sex and type of school.

**CONCLUSIONS:**

E-cigarette use is common among Peruvian adolescents, with dual use suggesting early nicotine dependence. Easy retail access and appealing flavors highlight regulatory gaps. Longitudinal studies are needed to clarify use trajectories and incidence and to inform effective public health and regulatory policies.

## INTRODUCTION

Tobacco smoking is a major risk factor for disability, chronic diseases, multiple cancers and premature mortality^1^. Globally, tobacco use constitutes a public health challenge, causing more than seven million preventable deaths each year^2^. Although control strategies have contributed to an 8.7-percentage-point reduction in tobacco use between 2007 and 2023^3^, new devices have emerged on the market as substitutes for conventional cigarettes^4^. These devices, commonly referred to as electronic nicotine (ENDS) and non-nicotine (ENNDS) delivery systems, including e-cigarettes, nicotine pouches and heated tobacco products, have been introduced in high-income countries since 2007^5^. Their uptake has been driven by perceptions of reduced harm compared with combustible cigarettes and their potential role in supporting smoking cessation^6^. However, existing evidence suggests that the use of e-cigarettes may be associated with adverse effects on respiratory, cardiovascular, neurological, gastrointestinal and oral health^7^.

In recent years, the use of e-cigarettes has increased markedly among adolescents and young adults, including those without a prior history of conventional cigarette smoking^8^. Globally, an estimated 16.8% of young people report ever using e-cigarettes and 4.8% report current use, although prevalence varies by sex, region and sociocultural context^9-11^. E-cigarettes are portable, battery-powered devices that replicate the behavioral and physiological features of conventional cigarettes. They deliver nicotine, often in its protonated form, together with solvents, flavorings and other toxic compounds that may independently or synergistically harm health and increase addictive potential^7,12,13^. Adolescents are particularly at risk, as exposure during this critical developmental stage may be associated with medium- and long-term health effects and an increased risk of subsequent conventional cigarette use^14,15^. Recent increases in dual use (i.e. concurrent smoking cigarettes and e-cigarettes) further indicate escalating nicotine dependence and underscore the urgency of public health action^16^.

In response to the increasing use of e-cigarettes among adolescents, the World Health Organization has reinforced its MPOWER strategy (Monitor tobacco use and prevention policies, Protecting people from tobacco smoke, Offering help to quit tobacco use, Warning about the dangers of tobacco, Enforcing bans on advertising, promotion and sponsorship and Raising taxes on tobacco)^3^. The latest report indicates that 133 countries regulate e-cigarettes in some form, including sales bans, health warnings and advertising restrictions, etc.^3^. However, many countries still lack regulation or systematic monitoring of e-cigarette use^3^. Adolescents remain a critical focus of these efforts, as targeted advertising, perceptions of reduced harm and the widespread availability of appealing flavors continue to drive uptake in this age group^17^. In Latin America and the Caribbean, available studies from Guatemala, Mexico, Ecuador, Colombia and Argentina estimate an overall prevalence of e-cigarette use of 18.9%^18^. Within the region, current use ranges from 2.9% in Guatemala to 15.8% among Colombian adolescents, underscoring substantial variation across countries^18^. Although several Latin American countries have reported data on e-cigarette prevalence, Peru lacks such evidence, limiting the ability to design context-specific policies^18^.

Therefore, this study addresses the research gap in Peru by analyzing population-based data on school-aged adolescents’ e-cigarette use patterns, including current, ever and dual use. In addition, the reasons for using and accessing e-cigarettes were assessed. Given the higher prevalence among boys^10^, sex-disaggregated analyses were undertaken.

## METHODS

### Study design and data sources

We conducted a secondary data analysis using data from the 2024 National survey on drug use in secondary school students, implemented in Peru by the National Commission for Development and Life Without Drugs (DEVIDA, in Spanish)^19^. The survey aimed to characterize current patterns of licit, illicit, and medicinal substance use among the school population. We collected data between August and September 2024 in urban areas with 30000 or more inhabitants^19^. The questionnaire used in the survey comprised 30 sections and a total of 138 questions. The instrument was piloted in two public schools and one private school in Lima, involving 353 students in first through fifth years of secondary education.

Although a formal psychometric validation was not performed, the questionnaire underwent a content review, internal testing of the optical answer sheet and a pilot study to ensure clarity, feasibility and consistency before national implementation^19^. The survey covered the 24 regions of the country and the Constitutional Province of Callao, with territorial representation across the three main geographical domains: coast, highlands and jungle.

### Study population and sample

The target population comprised students in the first to fifth years of secondary education, enrolled in public or private schools located in urban areas^19^. A stratified two-stage probabilistic sampling design, independent by region, was applied. In the first stage, schools were randomly selected, followed by the random selection of classes within each chosen school^19^. According to the official technical report, 406 schools (272 public and 134 private) across 72 cities were initially included in the sampling frame^19^. The final fieldwork collected data from 402 schools and 2004 classes, yielding a total of 45345 participants^19^. After applying expansion factors, these participants represent a total population of 1925076 secondary school students nationwide. The sampling design incorporated correction factors and adjustments for non-response rates^19^.

Of the 45345 participants included in the database, 45089 provided a valid response to the question on lifetime e-cigarette use. A total of 256 participants (0.56%) did not respond to this question and were therefore excluded from the analytical sample of the present study.

### Variables

For the present analysis, we considered outcomes related to e-cigarette and tobacco use, as well as sociodemographic variables to characterize the study population. The outcomes are defined in Table 1.

**Table 1:** Outcomes used to determine the prevalence and patterns of e-cigarette use among school-aged adolescents

*Outcomes*	*Description*
**E-cigarette**	
Age of initiation of e-cigarette use	Age (in years) at first e-cigarette use
Ever e-cigarette user	Use of an e-cigarette at least once in lifetime (yes, no)
12-month e-cigarette user	Use of an e-cigarette in the past 12 months (yes, no)
Frequency of e-cigarette	Number of days of e-cigarette use in the past 12 months, classified as 1–5, 6–19, or ≥20 days
Current e-cigarette user	Use of an e-cigarette in the past 30 days (yes, no)
Nicotine content in the e-cigarette	Whether the e-cigarette used contained nicotine (yes, no)
**Conventional cigarette**	
Age of initiation of conventional cigarette use	Age (in years) at first conventional cigarette use
Frequency of conventional cigarette	Number of days of conventional cigarette use in the past 12 months, classified as 1–5, 6–19, or ≥20 days
**Dual use**	
Dual user in the past 30 days	Concurrent use of e-cigarettes and conventional cigarettes in the past 30 days (yes, no)
Dual user in the past 12 months	Concurrent use of e-cigarettes and conventional cigarettes in the past 12 months (yes, no)
**Other**	
Sources of access	Reported sources of e-cigarette acquisition: corner stores/neighborhood shops, convenience stores, supermarkets, liquor stores, gas station shops, specialized shops, your own home, friends’ homes, relatives’ homes, home delivery
Motivations for e-cigarette use	Reported reasons for e-cigarette use: easy to carry, ease of use, because of the flavor, healthier alternative to conventional cigarettes, less odor than conventional cigarettes, curiosity, influence of friends or family, to manage stress or anxiety, more socially accepted than smoking conventional cigarettes

### Sociodemographic variables

Sociodemographic variables included age, recorded in completed years at the time of the survey and categorized as 11–13, 14–16 and 17–21 years; school grade (first to fifth year of secondary education); and type of school (public or private). In addition, we recorded participants’ school shift (morning, afternoon, or full-day), geographical location (Metropolitan Lima or the rest of the country) and nationality (Peruvian, Venezuelan, or other). Prevalence estimates for e-cigarette and tobacco outcomes, as well as sociodemographic characteristics, were disaggregated by sex (boys, girls), as recorded through interviewer observation in the DEVIDA survey.

### Statistical analysis

Analyses were conducted using Stata/SE 18.0 (StataCorp LLC, College Station, TX, USA) and a p<0.05 was considered statistically significant. The *svyset* command for survey analysis was applied to account for the complex sampling design and sampling weights. Standard errors were estimated using the Taylor series linearization method and the centered option was used to handle primary sampling units with a single stratum.

Weighted means and standard deviations (SD) were calculated for age and proportions with 95% confidence intervals (CI) were estimated for categorical variables, both for the total sample and disaggregated by sex. Mean differences between groups and their 95% confidence intervals were calculated using the *lincom* command after adjusting for survey design. An additional analysis was conducted to assess access to and reasons for e-cigarette use by school type (public, private). Group comparisons were performed using a t-test for continuous variables and the Rao–Scott corrected chi-squared test to examine differences in use patterns. Subpopulation analyses were conducted with the *subpop* option to ensure valid estimates under the complex survey design.

## RESULTS

We included a total of 45089 respondents in the analysis. Of them, 50% were girls (n=22534) and the mean age was 14.4 years (SD=1.5). Most participants were in the first (21.3%) or second (21.7%) year of secondary school, attended public schools (68.0%) in the morning shift (62.3%) and 36.5% were located in Metropolitan Lima. There are sex differences in specific sociodemographic characteristics. Girls had a higher proportion of Venezuelan origin (p=0.003) and were more likely to enroll in full-day schools (p<0.001), whereas boys had a higher proportion aged 17–21 years (p<0.001) and were more likely to attend public schools (p<0.001). Further details of sociodemographic characteristics by sex are shown in Table 2.

**Table 2:** Sociodemographic characteristics of participants in the 2024 National Survey on Drug Use in Secondary School Students, Peru (N=45089)

*Characteristics*	*Total* *(n=45089)*		*Boys* *(n=22555)*		*Girls* *(n=22534)*		*p*
	*n*	*Weighted %* *(95% CI)*	*n*	*Weighted %* *(95% CI)*	*n*	*Weighted %* *(95 % CI)*	
**Age** (years), mean (SD)	45089	14.4 (1.5)	22555	14.5 (1.5)	22534	14.4 (1.5)	0.001
**Age** (years)							
11–13	13707	31.0 (30.4–31.7)	6662	30.0 (29.0–30.9)	7045	32.1 (31.2–33.0)	<0.001
14–16	28008	61.4 (60.7–62.1)	14017	61.8 (60.8–62.8)	13991	61.0 (60.0–61.9)	
17–21	3374	7.6 (7.2–8.0)	1876	8.2 (7.7–8.8)	1498	6.9 (6.5–7.4)	
**Nationality**							
Peruvian	43347	95.4 (95.1–95.7)	21637	95.2 (94.7–95.6)	21710	95.7 (95.2–96.1)	0.003
Venezuelan	656	2.1 (1.9–2.3)	311	2.0 (1.7–2.3)	345	2.2 (1.9–2.6)	
Other	1086	2.5 (2.3–2.7)	607	2.8 (2.5–3.2)	479	2.1 (1.8–2.4)	
**Grade**							
First	9458	21.3 (20.7–21.9)	4715	21.2 (20.4–22.1)	4743	21.4 (20.6–22.2)	0.085
Second	9434	21.7 (21.1–22.3)	4687	21.9 (21.1–22.8)	4747	21.5 (20.8–22.4)	
Third	9054	19.7 (19.2–20.3)	4463	19.5 (18.7–20.3)	4591	19.9 (19.1–20.7)	
Fourth	8929	18.7 (18.2–19.3)	4461	18.1 (17.4–18.9)	4468	19.3 (18.5–20.1)	
Fifth	8214	18.6 (18.0–19.1)	4229	19.2 (18.4–20.0)	3985	17.9 (17.2–18.7)	
**Type of school**							
Public	32675	68.0 (67.3–68.7)	16113	66.0 (64.9–67.0)	16562	70.0 (69.0–71.0)	<0.001
Private	12414	32.0 (31.3–32.7)	6442	34.0 (33.0–35.1)	5972	30.0 (29.0–31.0)	
**School shift**							
Morning	26020	62.3 (61.6–62.9)	13097	62.8 (61.9–63.7)	12923	61.7 (60.8–62.6)	<0.001
Afternoon	12431	25.9 (25.3–26.4)	6226	26.7 (25.9–27.5)	6205	25.1 (24.3–25.9)	
Full day	6638	11.9 (11.5–12.3)	3232	10.5 (10.0–11.0)	3406	13.2 (12.6–13.8)	
**Location**							
Metropolitan Lima	4011	36.5 (35.7–37.3)	1984	36.5 (35.3–37.6)	2027	36.6 (35.5–37.7)	0.885
Rest of the country	41078	63.5 (62.7–64.3)	20571	63.5 (62.4–64.7)	20507	63.4 (62.3–64.5)	

Percentages and 95% confidence intervals are weighted and account for the complex survey design. Group comparisons by sex were performed using Student’s t-test for continuous variables and Rao–Scott chi-squared test for categorical variables.

In the overall sample, the mean age of initiation was 14.9 years (SD=1.5) for conventional cigarette use and 14.9 years (SD=1.4) for e-cigarette use. When disaggregated by sex, girls had a younger mean age of initiation for conventional cigarette use (14.7 [SD=1.4] vs 15.0 [SD=1.6]; mean age difference: 0.28; 95% CI: 0.18–0.39) and for e-cigarette use (14.7 [SD=1.3] vs 15.0 [SD=1.4]; mean age difference: 0.26; 95% CI: 0.16–0.36) compared with boys. The prevalence of ever e-cigarette use was 16.0% (95% CI: 15.5–16.6). This prevalence was higher among girls than among boys, with a 2.1 percentage-point difference (17.1% vs 15.0%, p<0.001). In addition, the prevalence of 12-month e-cigarette use was 10.9% (95% CI: 10.5–11.3), with a higher prevalence among girls than among boys (11.5% vs 10.3%, p=0.015). For this outcome, most participants (71.6%) reported e-cigarette use on 1–5 days in the past 12 months, whereas a smaller proportion reported use on ≥20 days. No differences were observed by sex (Table 3).

**Table 3:** Prevalence, age of initiation, and patterns of e-cigarette and conventional cigarette use among Peruvian secondary school students, 2024 (n=45089)

*Characteristics*	*Total* *(n=45089)*		*Boys* *(n=22555)*		*Girls* *(n=22534)*		*p*
	*n*	*Weighted %* *(95% CI)*	*n*	*Weighted %* *(95% CI)*	*n*	*Weighted %* *(95 % CI)*	
**Age of initiation of e-cigarette use** (years), mean (SD)	6493	14.9 (1.4)	3159	15.0 (1.4)	3334	14.7 (1.3)	<0.001
**Age of initiation of conventional cigarette use** (years), mean (SD)	5979	14.9 (1.5)	3228	15.0 (1.6)	2751	14.7 (1.4)	<0.001
**Ever e-cigarette user**	6493	16.0 (15.5–16.6)	3159	15.0 (14.3–15.8)	3334	17.1 (16.3–17.8)	<0.001
**12-month e-cigarette user**	4416	10.9 (10.5–11.3)	2176	10.3 (9.7–11.0)	2240	11.5 (10.8–12.1)	0.015
**Frequency of use** (days)							
1–5	3127	71.6 (69.6–73.6)	1507	70.5 (67.5–73.3)	1620	72.6 (69.8–75.2)	0.199
6–19	568	12.7 (11.3–14.2)	268	12.2 (10.2–14.5)	300	13.1 (11.2–15.2)	
≥20	666	15.7 (14.2–17.4)	367	17.3 (15.0–19.8)	299	14.3 (12.3–16.7)	
**Current e-cigarette user**	2779	6.9 (6.6–7.3)	1398	6.8 (6.3–7.3)	1381	7.0 (6.5–7.6)	0.508
**Type of e-cigarette**							
No nicotine	1225	45.3 (42.5–48.1)	621	45.7 (41.8–49.6)	604	44.9 (41.0–48.9)	0.802
With nicotine	1365	54.7 (51.9–57.5)	691	54.3 (50.4–58.2)	674	55.1 (51.1–59.0)	
**Dual user in the past 12 months**	2178	5.1 (4.8–5.4)	1190	5.4 (5.0–5.9)	988	4.8 (4.4–5.3)	0.057
**Frequency of e-cigarette use** (days)							
1–5	1489	70.0 (67.1–72.7)	791	68.5 (64.6–72.2)	698	71.6 (67.4–75.6)	0.529
6–19	297	12.2 (10.5–14.2)	165	12.8 (10.4–15.6)	132	11.5 (9.1–14.4)	
≥20	363	17.8 (15.6–20.4)	212	18.7 (15.7–22.1)	151	16.8 (13.5–20.7)	
**Frequency of cigarette use** (days)							
1–5	1453	69.1 (66.2–71.9)	762	65.4 (61.3–69.2)	691	73.4 (69.1–77.4)	0.023
6–19	274	13.2 (11.3–15.5)	153	14.7 (11.9–18.1)	121	11.5 (9.0–14.6)	
≥20	368	17.6 (15.4–20.1)	240	19.9 (16.9–23.2)	128	15.0 (11.9–18.9)	
**Dual user in the past 30 days**	1164	2.7 (2.4–2.9)	653	3.0 (2.6–3.3)	511	2.3 (2.1–2.6)	0.005
**Type of e-cigarette**							
No nicotine	446	38.9 (35.0–42.9)	255	39.4 (34.1–44.9)	191	38.2 (32.5–44.3)	0.773
With nicotine	675	61.1 (57.1–65.0)	377	60.6 (55.1–65.9)	298	61.8 (55.7–67.5)	

Percentages and 95% confidence intervals are weighted and account for the complex survey design. Group comparisons by sex were performed using Student’s t-test for continuous variables and Rao–Scott chi-squared test for categorical variables.

For current e-cigarette use, the prevalence was 6.9% (95% CI: 6.6–7.3) in the overall sample, with no differences by sex. Of these users, more than half reported using nicotine-containing e-cigarettes (54.7%). Moreover, the prevalence of dual use in the last 12 months (i.e. concurrent use of cigarette smoking and e-cigarette) was 5.1% (95% CI: 4.8–5.4). For this outcome, the most common frequency of use for conventional cigarettes and e-cigarettes was 1–5 days (69.1% and 70.0%, respectively), whereas a smaller proportion reported use on ≥20 days (17.8% and 17.6%, respectively). No differences were observed by sex in the pattern of dual use or in the frequency of use. In the past 30 days, the prevalence of dual use was 2.7% (95% CI: 2.4–2.9), with a higher proportion of students reporting nicotine-containing e-cigarette use (61.1% vs 38.9%). Boys had a higher prevalence of this pattern compared with girls (3.0% vs 2.3%, p=0.005), whereas the proportion of nicotine-containing e-cigarette use was similar in both sexes (Table 3).

**Figure 1:**
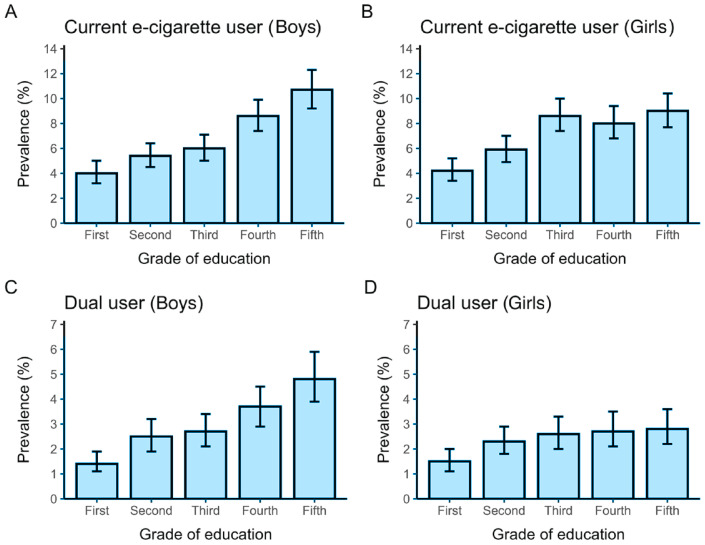
Prevalence of current e-cigarette users and dual users in the last 30 days among secondary school students in Peru, 2024: A) Current e-cigarette use among boys; B) Current e-cigarette use among girls; C) Dual use (cigarettes and electronic cigarettes) among boys; D) Dual use among girls. Bars represent

Sociodemographic characteristics for each outcome are shown in Supplementary file Table S1. The mean age was 14.9 years for ever, 12-month and current e-cigarette use and 15.0 years for dual use patterns. Dual use patterns included a higher proportion of adolescents aged 17–21 years. Across all outcomes, more than 90% of participants were Peruvian. Most adolescents attended public schools, predominantly in the morning shift and 30–40% were located in Metropolitan Lima. The distribution across grades of secondary education was broadly similar, with proportions gradually increasing from the first to the fifth grade across all outcomes. When the prevalence of current e-cigarette use and dual use in the past 30 days was examined by education level, a clear upward pattern was observed among males, whereas no consistent pattern was apparent among females (Figure 1).

Regarding access to e-cigarettes, the main sources were convenience stores (29.7%), corner or neighborhood shops (18.1%) and friends’ homes (11.5%). Other sources included liquor stores (9.6%), supermarkets (8.6%) and specialized shops (7.6%). When disaggregated by sex, the pattern of access differed. For both boys and girls, the main sources were convenience stores (28.9% in boys vs 30.5% in girls) and corner or neighborhood shops (18.9% in boys vs 17.4% in girls). However, boys more frequently obtained e-cigarettes from liquor stores (11.0% in boys vs 8.3% in girls), whereas girls more often reported access through friends’ homes (8.4% in boys vs 14.5% in girls). The main reasons for e-cigarette use in the overall sample were the flavor of e-liquids (30.8%), curiosity (20.4%) and managing stress and anxiety (12.9%). Other reasons included that the devices were easy to carry (9.0%), easy to use (8.6%) and perceived as a healthier alternative to conventional cigarettes (7.3%). Among both boys and girls, the main reasons for e-cigarette use were flavor (31.3% in boys vs 30.4% in girls) and curiosity (18.8% in boys vs 22.0% in girls). Boys reported using e-cigarettes because they were easy to carry (12.2% in boys vs 5.9% in girls) and perceived as a healthier alternative to conventional cigarettes (9.1% in boys vs 5.2% in girls), whereas girls more frequently cited stress and anxiety management (8.2% in boys vs 17.5% in girls) (Table 4).

**Table 4:** Sources of access to e-cigarettes and motivations for their use in the past 30 days among Peruvian secondary school students, 2024

*Characteristics*	*Total*		*Boys*		*Girls*		*p*
	*n*	*Weighted %* *(95% CI)*	*n*	*Weighted %* *(95% CI)*	*n*	*Weighted %* *(95 % CI)*	
**Sources of access to e-cigarettes**	2519		1278		1241		
Corner stores/neighborhood shops	409	18.1 (15.9–20.6)	214	18.9 (15.7–22.6)	195	17.4 (14.5–20.7)	0.005
Convenience stores	630	29.7 (27.1–32.4)	316	28.9 (25.4–32.8)	314	30.5 (26.8–34.5)	
Supermarkets	245	8.6 (7.3–10.2)	141	9.8 (8.0–12.0)	104	7.4 (5.6–9.8)	
Liquor stores	261	9.6 (8.0–11.5)	154	11.0 (8.8–13.7)	107	8.3 (6.1–11.0)	
Gas station shops	95	3.7 (2.8–4.8)	59	4.6 (3.3–6.5)	36	2.7 (1.8–4.2)	
Specialized shops	262	7.6 (6.4–9.0)	147	8.4 (6.7–10.6)	115	6.8 (5.3–8.7)	
Your own home	74	2.9 (2.0–4.0)	32	2.0 (1.2–3.2)	42	3.7 (2.4–5.8)	
Friends’ homes	318	11.5 (9.4–13.6)	108	8.4 (6.4–11.0)	210	14.5 (12.0–17.4)	
Relatives’ homes	45	1.8 (1.1–2.7)	21	1.5 (0.7–2.9)	24	2.1 (1.3–3.6)	
Home delivery	180	6.5 (5.3–8.0)	86	6.4 (4.8–8.6)	94	6.6 (5.0–8.7)	
**Motivations for e-cigarette use**	2541		1289		1252		
Easy to carry	242	9.0 (7.4–10.8)	171	12.2 (9.6–15.4)	71	5.9 (4.3–7.9)	<0.001
Ease of use	214	8.6 (7.1–10.3)	112	7.9 (5.9–10.5)	102	9.2 (7.1–11.9)	
Because of the flavor	782	30.8 (28.3–33.5)	403	31.3 (27.8–35.0)	379	30.4 (26.8–34.3)	
Healthier alternative to conventional cigarettes	189	7.3 (5.8–8.7)	111	9.1 (7.0–11.8)	78	5.2 (3.8–7.0)	
Less odor than conventional cigarettes	162	5.9 (4.8–7.3)	97	6.9 (5.2–9.3)	65	4.9 (3.6–6.7)	
Curiosity	498	20.4 (18.1–22.8)	225	18.8 (15.8–22.2)	273	22.0 (18.7–25.6)	
Influence of friends or family	87	3.4 (2.6–4.5)	37	3.1 (2.1–4.6)	50	3.7 (2.6–5.4)	
To manage stress or anxiety	311	12.9 (11.0–15.0)	96	8.2 (6.2–10.7)	215	17.5 (14.5–20.9)	
More socially accepted than smoking conventional cigarettes	56	1.9 (1.3–2.7)	37	2.5 (1.6–4.0)	19	1.3 (0.8–2.3)	

Percentages and 95% confidence intervals are weighted and account for the complex survey design. Group comparisons by sex were performed Rao–Scott chi-squared test.

Access to, and reasons for, e-cigarette use by type of school (public, private) are presented in Supplementary file Table S2. No differences were found in access to e-cigarettes. In both groups, the main sources were convenience stores, corner or neighborhood shops and friends’ homes. Regarding reasons for e-cigarette use, adolescents from public schools more often reported flavor (33.0% in public vs 26.1% in private) and ease of carrying (10.2% in public vs 6.3% in private) as the main motives, whereas those from private schools more frequently cited curiosity (17.9% in public vs 25.8% in private), managing stress and anxiety (12.1% in public vs 14.6% in private) and the influence of friends and family (2.1% in public vs 6.3% in private).

## DISCUSSION

This study examined the prevalence of e-cigarette use patterns among 45089 school-aged adolescents in Peru. Across all outcomes, the proportion of adolescents increased steadily from first to fifth grade. Among outcomes assessed in the past 30 days, most adolescents reported consumption of nicotine-containing e-cigarettes. Sex-disaggregated analyses showed that girls initiated e-cigarette use at an earlier age and had a higher prevalence of ever and 12-month use, whereas boys had a higher prevalence of dual use in the past 30 days. On the other hand, we explored the main sources of access and reasons for e-cigarette use. Convenience stores, corner or neighborhood shops and friends’ homes were the predominant points of access, while the most frequently reported reasons were the flavor of e-liquids, curiosity and managing stress and anxiety. Among boys, easy to carry and the perception of being a healthier alternative to conventional cigarettes were more frequently reported, whereas girls more often cited stress and anxiety management. In addition, students from public schools more often reported flavor and ease of carrying as the main motives, whereas those from private schools more frequently cited curiosity, managing stress and anxiety and the influence of friends and family.

These findings are consistent with the existing literature on e-cigarette use patterns. For example, the prevalence of ever and current e-cigarette use in our study was similar to estimates from analyses of Global Youth Tobacco Survey data, which reported prevalence in Peru of 13.4% and 6.9%, respectively^20^. Moreover, when comparing our results with data from 13 countries in Latin America and the Caribbean, we found that the prevalence of ever and current e-cigarette use among Peruvian adolescents falls within the regional range for both patterns of use^18^. Although existing evidence suggests that both patterns are more common in boys than in girls^10^, our study observed that girls had a higher prevalence of ever and 12-month e-cigarette use. This finding contrasts with previously reported sex differences in cigarette smoking^1^. However, existing research suggests that flavored e-cigarette marketing may disproportionately attract girls, which could help explain our finding, further supported by their earlier age of initiation compared with boys in our study^21^. Among adolescents who reported current e-cigarette use, most used devices containing nicotine. Since nicotine is one of the most widely used drugs worldwide, its addictive potential and associated health harms, particularly among adolescents, make e-cigarette use in this age group an emerging public health concern that should be addressed in the development of health policies^14,23^. In line with this, our study found that the mean age of initiation of e-cigarette use was 14.9 years. Such early initiation increases the likelihood of lifelong nicotine dependence and subsequent cigarette smoking, which remains one of the leading contributors to disability and premature mortality^15^. An additional key finding was that the proportion of adolescents reporting each outcome, rose progressively from the first to the fifth grade, a trend consistent with results from other settings^23,24^. As students move to higher grades in secondary school, their growing social interactions with peers, heightened autonomy and reduced perception of risk, may contribute to this upward pattern^25^.

One of the emerging patterns in this population is the concurrent use of conventional cigarettes and e-cigarettes (i.e. dual use). This pattern is of particular public health importance, as adolescents are simultaneously exposed to the toxic health effects of conventional cigarettes and the harmful effects of e-cigarettes^26^. The results related to dual use in the past 30 days are comparable with studies using Global Youth Tobacco Survey data, in which the prevalence of this pattern ranges from 0.1% to 13.6%^11^. At the regional level, our estimates are lower than those reported in European countries and South Africa^27,28^, underscoring that the prevalence of dual use varies by region. Moreover, our results show that most adolescents who reported dual use in the past 30 days used nicotine-containing e-cigarettes. Considering this finding, dual use is likely to be associated not only with greater nicotine dependence but also with additional health risks arising from the concurrent use of both products. Existing research reports that dual use is associated with increased odds of cardiovascular disease, metabolic dysfunction, asthma, chronic obstructive pulmonary disease and oral disease compared with individuals who smoke cigarettes only or do not use either product^29^. Therefore, understanding the prevalence of this pattern and the fact that boys show a higher prevalence may help inform targeted public health strategies for controlling tobacco and e-cigarette use.

The main sources of access to e-cigarettes were convenience stores, corner or neighborhood shops and friends’ homes. Other sources identified in our study included liquor stores, supermarkets and specialized shops. These findings are consistent with existing evidence. A study conducted in the United States reported that the main sources of access to e-cigarettes among adolescents aged 13–17 years were retail stores, vape shops, gas stations and convenience stores^30^. Moreover, this age group often obtained e-cigarettes from people they knew^30^. Although the sale of these devices is legally restricted to adults in Peru, the main sources of access were convenience and neighborhood stores, which might reflect a lack of age verification in these establishments^31^. In addition, our study highlights sex differences. Boys were more likely to access these devices independently, whereas girls more often obtained them through people they knew^32^. On the other hand, the main reasons for using e-cigarettes were the flavor of e-liquids, curiosity and managing stress and anxiety. Similar results have been reported among US adolescents, who use e-cigarettes for various reasons such as curiosity, product characteristics and the influence of friends or family^33^. In a recent systematic review, similar motives were reported, including low perceived risk and appealing flavors^17^. These motives differed by sex and by type of school (public vs private) in our study. With regard to sex, previous research shows that boys are more likely to perceive e-cigarettes as less harmful than conventional cigarettes, which may partly explain their greater susceptibility to use^34^. In addition, the differences observed by school type suggest that contextual and social environments shape adolescents’ motives. Students in public schools more often emphasized product characteristics, whereas those in private schools highlighted social and emotional drivers. These reasons appear consistent across contexts, underscoring the need for targeted public health interventions and regulatory measures to reduce the appeal of e-cigarettes among adolescents. School-based control policies, irrespective of school type, have also demonstrated benefits in lowering e-cigarette use, reinforcing the value of multi-level strategies^35^.

From a health policy perspective, our findings provide an evidence base to adapt and strengthen existing strategies, although longitudinal studies are still needed to better understand use trajectories and long-term outcomes. In Peru, governmental institutions have introduced control measures for e-cigarettes, including bans on sales to minors, restrictions on use in enclosed public spaces and public transport, and prohibitions on advertising^36^. However, these measures were implemented without context-specific data and their effectiveness may therefore be limited. Based on our findings, these strategies should be reinforced. Preventive mechanisms are needed to control the prevalence of e-cigarette and dual use. Strategies to be considered include stricter enforcement at key points of access, such as convenience stores and neighborhood shops, to ensure compliance with age-of-sale restrictions. In addition, school- and community-based campaigns should deliver tailored educational programs on the risks of nicotine and e-cigarettes, using age-appropriate and engaging strategies. These programs could be integrated into school curricula, reinforced through extracurricular activities and complemented by parental involvement at home. Moreover, a crucial aspect of access to and use of e-cigarettes in Peru could be addressed through the implementation of specific taxes on e-cigarettes, similar to those applied to conventional cigarettes. This measure has proven to be an important component of MPOWER strategies to reduce tobacco consumption^3^. Therefore, strengthening fiscal and educational policies could play a key role in curbing e-cigarette use among adolescents.

### Limitations

The results of our study are not without limitations. First, the descriptive design does not allow for the establishment of associations between study variables and instead aims to describe patterns of e-cigarette use among Peruvian adolescents using a population-based survey. Second, device-related characteristics such as generation, nicotine concentration, flavor of e-liquids, number of vaping episodes per day and weekly frequency of use were not available, limiting further characterization of use patterns in the study population. Third, patterns of e-cigarette use are subject to misclassification bias, as all data were self-reported. Nevertheless, reliance on past 30-day outcomes reduces bias and increases the reliability of these measures. Fourth, the use of self-reported behaviors might also be subject to social desirability bias. Fifth, we did not estimate age-adjusted prevalence because most participants were aged 12–17 years and only a small proportion were aged 11 years or 18–21 years. As data were collected exclusively from schools in urban areas, the representativeness of populations in towns with fewer than 30000 inhabitants and in rural settings may be limited. However, little is known about the prevalence of e-cigarette use in rural or remote areas, particularly given the challenges these settings face in terms of availability and access to e-cigarettes^37^. Furthermore, our findings are derived from a single country with distinct cultural and behavioral characteristics, which restricts the generalizability of the results to other contexts. An additional outcome evaluated in our study was dual use in the past 12 months. Although this outcome showed a higher prevalence than dual use in the past 30 days, it has some limitations. Because of the longer timeframe, the cross-sectional design makes it difficult to determine whether adolescents used both products concurrently and the measure might be subject to misclassification bias. Nevertheless, this outcome could be further explored in longitudinal studies to assess a potential dose-response relationship between the duration of dual use and the development of several diseases.

## CONCLUSIONS

This population-based study of Peruvian adolescents found that e-cigarette use begins at an early age. Sex differences were evident, with ever and 12-month use more frequent among girls, whereas dual use in the past 30 days was more common among boys. Across all outcomes, the proportion of adolescents reporting e-cigarette use increased progressively with advancing secondary school grades. The main sources of access were convenience stores, neighborhood shops and friends’ homes, while the most frequently reported reasons were the flavor of e-liquids, curiosity and managing stress and anxiety. Differences in reasons for use by sex and type of school underscore the need for tailored prevention strategies. Existing measures should be reinforced through stricter enforcement at points of sale, school- and community-based educational campaigns and the introduction of specific taxes on e-cigarettes. In parallel, longitudinal studies are needed to assess use trajectories and long-term health outcomes.

## Supplementary Material



## Data Availability

The DEVIDA data supporting this research are available upon request from the following source: https://www.transparencia.gob.pe/reportes_directos/pep_transparencia_acceso_informacion.aspx?id_entidad=11793&id_tema=49&cod_rueep=0&ver=
