# Supplementary material for: Prevalence and patterns of e-cigarette use among school-aged adolescents in Peru, 2024
Source: Tob Induc Dis. 2026 Jun 5;24:10.18332/tid/218168. doi: 10.18332/tid/218168 (PMC13241958; doi:10.18332/tid/218168)
Supplement: Supplementary file 1 [file TID-24-80-s1.pdf]

Table S1. Sociodemographic characteristics of adolescents by patterns of e-cigarette and dual use, Peru 2024

Charcateristic	Ever e-cigarette user		12-month e-cigarette user		Current e-cigarette user		Dual user in the past 12 months		Dual user in the past 30 days	
	n	Weighted % (95% CI)	n	Weighted % (95% CI)	n	Weighted % (95% CI)	n	Weighted % (95% CI)	n	Weighted % (95% CI)
Age in years (mean, SD)	6493	14.9 (1.4)	4416	14.9 (1.4)	2779	14.9 (1.4)	2178	15.0 (1.4)	1164	15.0 (1.5)
Age group (years)										
11 to 13	1158	19.3 (17.9-20.8)	764	18.1 (16.4-19.9)	500	19 (16.9-21.4)	368	16 (13.9-18.4)	201	16.6 (13.9-19.6)
14 to 16	4645	70.0 (68.4-71.7)	3184	70.9 (68.9-72.8)	1977	69.8 (67.2-72.3)	1537	71.7 (68.9-74.3)	810	69.8 (66.1-73.3)
17 to 21	690	10.7 (9.6-11.8)	468	11 (9.8-12.4)	302	11.1 (9.6-12.9)	273	12.3 (10.6-14.3)	153	13.6 (11.1-16.6)
Nationality										
Peruvian	6121	93.5 (92.5-94.4)	4151	93.4 (92.3-94.4)	2593	92.8 (91.3-94.1)	2030	92.6 (90.9-94.1)	1074	92 (89.6-94)
Venezuelan	164	3.5 (2.8-4.3)	115	3.3 (2.6-4.2)	80	3.5 (2.6-4.7)	64	3.9 (2.8-5.5)	41	4.5 (2.9-6.8)
Other	208	3.0 (2.5-3.7)	150	3.3 (2.6-4.1)	106	3.7 (2.8-4.8)	84	3.5 (2.6-4.6)	49	3.5 (2.5-4.8)
Grade of education										
First	729	12.4 (11.3-13.7)	475	11.4 (10.1-12.9)	334	12.6 (10.9-14.6)	235	10.1 (8.4-12.2)	140	11.7 (9.5-14.4)
Second	1156	18.5 (17.2-20.0)	766	17.6 (16-19.3)	491	17.6 (15.7-19.8)	379	18.4 (16.1-21)	214	19.4 (16.3-22.8)
Third	1332	20.0 (18.6-21.4)	912	19.8 (18.1-21.5)	578	20.8 (18.7-23.1)	433	19.7 (17.3-22.2)	232	19.3 (16.3-22.8)
Fourth	1601	23.7 (22.2-25.3)	1113	24.5 (22.7-26.5)	664	22.4 (20.2-24.8)	526	23.8 (21.3-26.6)	268	22.4 (19.3-25.9)
Fifth	1675	25.4 (23.9-27.0)	1150	26.7 (24.8-28.7)	712	26.5 (24.1-29.1)	605	28 (25.3-30.8)	310	27.2 (23.7-31)
Type of School										
Public	4390	66.6 (64.7-68.4)	2924	66.3 (64.1-68.4)	1902	67.9 (65.1-70.6)	1527	69.7 (66.6-72.7)	862	77 (73.5-80.2)
Private	2103	33.4 (31.6-35.3)	1492	33.7 (31.6-35.9)	877	32.1 (29.4-34.9)	651	30.3 (27.3-33.4)	302	23 (19.8-26.5)
School shift										
Morning	3673	61.2 (59.5-62.9)	2497	60.6 (58.5-62.6)	1586	62.1 (59.6-64.6)	1169	58.9 (55.9-61.7)	611	57.7 (53.8-61.5)
Afternoon	1900	26.9 (25.5-28.4)	1278	27.1 (25.4-29)	795	26.1 (24-28.4)	684	29.2 (26.7-31.9)	373	30 (26.7-33.7)
Full day	920	11.8 (10.8-12.9)	641	12.3 (11.1-13.7)	398	11.8 (10.3-13.4)	325	11.9 (10.3-13.7)	180	12.3 (10.3-14.6)
Geographic Location										
Metropolitan Lima	719	40.2 (38.2-42.3)	488	40 (37.6-42.5)	315	40.1 (37.1-43.2)	211	36.3 (32.8-39.9)	107	33 (28.5-37.9)

Rest of the country	5774	59.8 (57.7-61.8)	3928	60 (57.5-62.4)	2464	59.9 (56.8-62.9)	1967	63.7 (60.1-67.2)	1057	67 (62.1-71.5)

Ever e-cigarette user: Use of an e-cigarette at least once in lifetime (yes/no).
12-month e-cigarette user: Use of an e-cigarette in the past 12 months (yes/no).
Current e-cigarette user: Use of an e-cigarette in the past 30 days (yes/no).
Dual user in the past 30 days: Concurrent use of e-cigarettes and conventional cigarettes in the past 30 days (yes/no).
Dual user in the past 12 months: Concurrent use of e-cigarettes and conventional cigarettes in the past 12 months (yes/no).
Percentages and 95% confidence intervals are weighted and account for the complex survey design.
Group comparisons by sex were performed using Student’s t-test for continuous variables and Rao–Scott chi-square test for categorical variables. Abbreviations: n = unweighted frequency; CI = confidence interval; SD = standard deviation.

Table S2. Sources of access to and motivations for e-cigarette use among Peruvian adolescents, by type of school (public vs private), 2024

Characteristics	Public school		Private school		p-value
	n	Weighted % (95% CI)	n	Weighted % (95% CI)	
Sources of access to e-cigarettes					
Corner stores / neighbourhood shops	306	18.8 (16.3-21.5)	103	16.6 (12.4-21.9)	0.101
Convenience stores	437	31.2 (28.2-34.5)	193	26.3 (21.8-31.5)	
Supermarkets	189	9.5 (7.9-11.4)	56	6.5 (4.4-9.7)	
Liquor stores	187	9.3 (7.7-11.2)	74	10.3 (6.9-15.1)	
Gas station shops	64	3.4 (2.5-4.6)	31	4.3 (2.6-7.1)	
Specialised shops	156	7.0 (5.7-8.5)	106	9.1 (6.8-12.1)	
Your own home	48	2.6 (1.8-3.9)	26	3.4 (1.8-6.2)	
Friends' homes	209	10.9 (9.1-13.2)	109	12.6 (9.6-16.5)	
Relatives' homes	28	1.9 (1.1-3.2)	17	1.4 (0.7-3.0)	
Home delivery	117	5.3 (4.2-6.7)	63	9.3 (6.5-13.0)	
Motivations for e-cigarette use					
Easy to carry	201	10.2 (8.5-12.3)	41	6.3 (3.6-10.9)	<0.001
Ease of use	167	9.7 (7.9-11.9)	47	6.0 (3.7-9.6)	
Because of the flavour	551	33.0 (30.0-36.1)	231	26.1 (21.7-31.1)	
Healthier alternative to traditional cigarettes	123	7.0 (5.5-9.0)	66	7.3 (5.2-10.2)	
Less odour than traditional cigarettes	101	5.9 (4.5-7.7)	61	6.0 (4.3-8.5)	
Curiosity	314	17.9 (15.6-20.5)	184	25.8 (21.0-31.3)	
Influence of friends or family	40	2.1 (1.4-3.1)	47	6.3 (4.3-9.1)	
To manage stress or anxiety	218	12.1 (10.2-14.4)	93	14.6 (10.7-19.4)	
More socially accepted than smoking traditional cigarettes	37	1.3 (1.3-2.3)	19	1.5 (0.9-2.5)	

Percentages and 95% confidence intervals are weighted and account for the complex survey design. Group comparisons were performed using Rao–Scott chi-square test.

Abbreviations: n = unweighted frequency; CI = confidence interval.

© 2026 Vargas-Fernández R. and Hernández-Vásquez A.
